# Complex Multilevel Modelling of the Individual, Household and Regional Level Variability in Predictors of Undernutrition among Children Aged 6–59 Months in Ethiopia

**DOI:** 10.3390/nu13093018

**Published:** 2021-08-29

**Authors:** Teshita Uke Chikako, Abdul-Aziz Seidu, John Elvis Hagan, Bright Opoku Ahinkorah

**Affiliations:** 1 Wondo Genet College of Forestry and Natural Resource, Hawassa University, Hawassa P.O. Box 05, Ethiopia; teshitauke@hu.edu.et; 2 Department of Population and Health, University of Cape Coast, Cape Coast TF0494, Ghana; abdul-aziz.seidu@stu.ucc.edu.gh; 3 College of Public Health, Medical and Veterinary Sciences, James Cook University, Townsville, QLD 4811, Australia; 4 Department of Estate Management, Takoradi Technical University, Takoradi P.O. Box 256, Ghana; 5 Department of Health, Physical Education and Recreation, University of Cape Coast, Cape Coast TF0494, Ghana; 6 Neurocognition and Action-Biomechanics-Research Group, Faculty of Psychology and Sport Sciences, Bielefeld University, Postfach 100131, 33501 Bielefeld, Germany; 7 Faculty of Health, School of Public Health, University of Technology Sydney, Sydney, NSW 2007, Australia; bright.o.ahinkorah@student.uts.edu.au

**Keywords:** children, DHS, Ethiopia, malnutrition, multilevel

## Abstract

Worldwide, ten and a half million children under five die every year, with 98% of these deaths in low- and middle-income countries, including Ethiopia. Undernutrition is a serious public health problem in Ethiopia and children are the most affected segments of the population. This study, therefore, sought to investigate the socio-economic, demographic, health and environmental factors associated with undernutrition among children aged 6–59 months in Ethiopia. Data were obtained from the 2016 Ethiopian Demographic and Health Survey. In this study, anthropometric data (height and weight) and other variables of 9461 children were measured. Descriptive statistics and multilevel logistic regression models were fitted. The descriptive results revealed that about 27.5% of the children aged between 6–59 months were undernourished. Place of residence, employment status of the mother, educational status of the mother, the mother’s nutritional status, age of the child, birth order of children, source of drinking water, diarrhea and fever among children in the two weeks before the survey were the most important factors associated with undernutrition among children aged 6–59 months in Ethiopia. The findings indicate that it is useful to support health care and food security programs in rural areas to directly address food insecurity and undernutrition problems of the poor and exposed communities in rural parts of the country. The education sector must increase mothers’ access to education in all areas to help identify the quality of healthcare and the required attention needed for their children. The health sector should increase their health education programs on the importance of exclusive breastfeeding.

## 1. Introduction

Malnutrition remains a major public health problem in low- and middle-income countries (LMICs) [1]. It is the most important risk factor for the load of disease affecting about 300,000 deaths per year directly and indirectly and accounting for more than half of all deaths in children [2]. Nowadays, child malnutrition is the most shocking problem challenging the majority of LMICs [3] and one of the most common reasons of child death in LMICs [4]. Malnutrition alone is accountable for over half of the losses in children under five in LMICs [5].

Malnutrition occurs when the body does not receive the appropriate quantity of nutrients that are essential to keep the organs and tissues healthy and operational [6,7]. A number of studies [8,9,10] indicate that children and females are the prime sufferers of malnutrition. Children are said to be malnourished if they are undernourished or over nourished, but most of the time malnutrition happens when people are undernourished [11,12]. The main causes for undernutrition, especially in children, are poverty, non-existence of food, frequent sicknesses, unsuitable suckling practices, absence of care and underprivileged cleanliness [12]. Inadequate nutrients in the first two years of life can cause a child’s body to become sluggish and can stunt intellectual growth for the rest of his or her life. A short period of insufficient nutrients, along with sickness or infection, can rapidly make a child seriously undernourished [13].

Poor nutrition at childhood obstructs a child’s bodily and intellectual development that eventually causes the malicious cycle of intergenerational undernutrition [14,15]. Undernutrition is a soundless killer that is under testified, under identified and, also, under arranged [16]. For every minute of each day, five children die due to undernutrition [17,18].

Reducing undernutrition in children under five remains major challenge in LMICs [18]. A projected 230 million children under five are understood to be habitually malnourished in LMICs [19]. Correspondingly, around 54% of losses among children of this age group are thought to be related to malnourishment in LMICs [20]. In sub-Saharan Africa, 41% of children under five were undernourished in 2016 and losses from malnourishment were rising on regular basis [20,21].

Results from the 2016 Ethiopian Demographic and Health Survey (EDHS) indicate that stunting (chronic undernutrition) and being underweight (chronic and acute undernourishment) was seen in 38% and 24% of children less than five years old, respectively [22]. Undernutrition in children is one of the most severe public health challenges in Ethiopia [23]. For example, almost one in every 17 babies born in Ethiopia (59 per 1000) will not live to rejoice its first anniversary, and one in every eleven children (88 per 1000) will die before its fifth birthday [23].

Even though the problem of child undernutrition in Ethiopia has been sufficiently recognized, the severity and the explanations behind it are quite scanty. There is also a discrepancy between studies concerning the predictors of undernutrition. This discrepancy might be because of the insertion and/or elimination of some variables. Estimations might also vary based on various variables and types of data and estimation methods. Even though these overall realities are clear, the detailed factors that lead to undernutrition in children under five in Ethiopia have received little research attention. Consequently, this paper aims to investigate the socio-economic, demographic, health and environmental factors associated with undernutrition among children aged 6–59 months in Ethiopia. The specific objectives of the study were:

1. To identify the socio-economic, demographic, health and environmental factors that lead to undernutrition among under five children in Ethiopia;
2. To examine the level of within-household and between-household differences in determinants of undernutrition of children under five and
3. To examine the level of within-regional and between-regional differences in determinants of undernutrition in children under five.

## 2. Materials and Methods

### 2.1. Source of Data

The study used data from the 2016 version of the EDHS on the malnourishment status of a child by assessing the height and weight of all children aged 6–59 month. The information was gathered to compute three key anthropometric pointers: weight-for-age (underweight), height-for-age (stunting) and weight-for-height (wasting). In the 2016 EDHS, out of 10,752 children under five only 9461 were measured for the anthropometric indices as well as the other related variables. As a result, the sample for the current study comprised 9461 children under five with complete anthropometric data. Details of the EDHS methodology and sampling has been published in the EDHS report [22] which is also available online at https://dhsprogram.com/publications/publication-fr328-dhs-final-reports.cfm?cssearch=351226_1 (accessed on 20 May 2021).

### 2.2. Variables of the Study

#### 2.2.1. Outcome Variables

In assessing the undernutrition status of children, the basic anthropometric measurements of weight and height were considered. The 2016 EDHS collected data on the nutritional status of children by measuring the weight and height of children under the age of five in all sampled households, regardless of whether their mothers were interviewed in the survey. Weight was measured with an electronic mother–infant scale (SECA 878 flat) designed for mobile use. Height was measured with a measuring board (Shorr Board^®^). Children younger than 24 months were measured lying down on the board (recumbent length), while standing height was measured for the older children. Since children’s weight and height changes with age, these measurements were transformed to Z-scores based on the National Centre for Health Statistics (NCHS) reference population recommended by the World Health Organization [22] (p. 186). The three indices of undernutrition were:

1. Stunting (Low height for age): Stunting is a reflection of the lifelong experience of child that is sometimes referred to us “chronic undernutrition ”.
2. Wasting (Low weight for height): Wasting is a reflection of the thinness of child. Thinness tends to respond to a more recent experience (food intake or disease). Wasting is sometimes referred to us “acute undernutrition”.
3. Underweight (Low weight for age): Underweight is a summary indication of the degree of stunting and wasting in a child because height deficits and thinness will both result in the child weighting less than normal for his or her age.

Therefore, those below −2 standard deviations of the NCHS median reference for height-for-age, weight-for-age and weight-for-height were considered as stunted, underweighted, and wasted, respectively. The response variable which is a measure of undernutrition is dichotomized as 1 if a child is undernourished (if a child is stunted, wasted or underweight) and 0 if normal/nourished.

#### 2.2.2. Explanatory Variables

The explanatory variables used in this study were grouped into demographic variables (sex of child, age of child, sex of household head, and birth order of the child), socio-economic variables (the mother’s education, employment status of the mother, wealth index, household size, place of residence, geographical region, and the mother’s body mass index), and health related variables (source of water supply, type of toilet facility, had diarrhea, and had fever). These factors were selected based on previous literature which found them to be related with malnourishment among children under five [1,6,7]. 

### 2.3. Data Analyses

Descriptive (frequencies and percentages) and multilevel analysis were carried out in this study. Multilevel analysis is used if the data have grouped structures, through elementary units at level 1 nested within groups at level 2, which successively nested within groups at level 3, and so on. The study considers three levels of hierarchy data (that is, a child is nested in the household and the household is nested in the region of the country). Therefore, in the data, regions (Reg) representing blocks of households (HH) are at level 3, households are at level 2 and individual children at level 1. Considering a dichotomous outcome variable *y_ijk_* ∼ Bernoulli (*π_ijk_*), then the logit link functions are given by:

$$\tau_{ijk}=\beta_{0}+\beta_{1}x_{ijk}+\beta_{2}x_{HH\left(jk\right)}+\beta_{3}x_{Reg\left(k\right)}+\varepsilon_{jk}+\varepsilon_{k}$$

where $\tau_{ijk}$ = $n\left(\frac{\pi_{ijk}}{1-\pi_{ijk}}\right)$, $\pi_{ijk}$ means the probability of the $i^{th}$ child in the $j^{th}$ household and the $k^{th}$ region were malnourished; i.e., $\pi_{ijk}$ = P($Y_{ijk}=1$), $x_{ijk}$ denotes the vector of the first-level variables, $x_{HH\left(jk\right)}$ denotes the vector of the second-level variables and $x_{Reg\left(k\right)}$ denotes the vector of the third-level predictor variables. In addition, $\beta_{1}$ denotes the vector of regression parameters for the first-level variables, $\beta_{2}$ denotes the vector of regression parameters for the second-level variables and $\beta_{3}$ denotes the vector of regression parameters for the third-level variables. $\varepsilon_{jk}$ denotes the random effect for the $j^{th}$ level cluster in the $k^{th}$ level cluster, and $\varepsilon_{k}$ denotes the random effect for the $k^{th}$ third-level cluster [24]. We accept that $\varepsilon_{jk}\sim \;N\left(0,\;\sigma_{HH}^{2}\right)\;\mathrm{and}\;\varepsilon_{k}\sim \;N\left(0,\;\sigma_{Reg}^{2}\right)$ are independent.

#### 2.3.1. Intercept-Only Model

This is the modest event of a multilevel model for a dichotomous outcome variable in which no explanatory variables at all were considered.

$$\tau_{ijk}=\beta_{0}+\varepsilon_{jk}+\varepsilon_{k}$$

where $\varepsilon_{jk}$ indicates the random part for the $j^{th}$ level group in the $k^{th}$ level group and $\varepsilon_{k}$ denotes the random effect for the $k^{th}$ third-level group.

#### 2.3.2. Random Intercept Model

In this model, the covariates are included and the intercept is the only random effect, which means the clusters vary with respect to the average value of the response variable. This model assumes the slopes are fixed.

$$\tau_{ijk}=\beta_{0}+\beta_{1}x_{ijk}+\beta_{2}x_{HH\left(jk\right)}+\beta_{3}x_{Reg\left(k\right)}+\varepsilon_{jk}+\varepsilon_{k}$$

where $x_{ijk}$ denotes the vector of the first-level variables, $x_{Com\left(jk\right)}$ denotes the vector of the second-level variables and $x_{Reg\left(k\right)}$ denotes the vector of the third-level predictor variables. In addition, $\beta_{1}$ denotes the vector of regression parameters for the first-level variables, $\beta_{2}$ denotes the vector of regression parameters for the second-level variables and $\beta_{3}$ denotes the vector of regression parameters for the third-level variables. $\varepsilon_{jk}$ denotes the random effect for the $j^{th}$ level cluster in the $k^{th}$ level cluster and $\varepsilon_{k}$ denotes the random effect for the $k^{th}$ third-level cluster [24].

#### 2.3.3. Random Coefficient Model

In this model, the coefficients of the explanatory variables are considered as random. Random coefficient models treat the covariate as well as the intercept as random variables that can explain unnoticed differences in the effects of predictor variables on the outcome variable.

$$\tau_{ijk}=\beta_{0}+\beta_{1}x_{ijk}+\beta_{2}x_{HH\left(jk\right)}+\beta_{3}x_{Reg\left(k\right)}+\varepsilon_{jk}+\varepsilon_{ojk}x_{ijk}+\varepsilon_{k}+\varepsilon_{ok}x_{ijk}$$

where $x_{ijk}$ denotes the vector of the first-level variables, $x_{HH\left(jk\right)}$ denotes the vector of the second-level variables and $x_{Reg\left(k\right)}$) denotes the vector of the third-level predictor variables. In addition, $\beta_{1}$ denotes the vector of regression parameters for the first-level variables, $\beta_{2}$ denotes the vector of regression parameters for the second-level variables and $\beta_{3}$ denotes the vector of regression parameters for the third-level variables. $\varepsilon_{jk}$ denotes the random effect for the $j^{th}$ level cluster in the $k^{th}$ level cluster and $\varepsilon_{k}$ denotes the random effect for the $k^{th}$ third-level cluster [24]. $\varepsilon_{ojk}$ and $\varepsilon_{ok}$ denote random slopes. The part $\beta_{0}+\beta_{1}x_{ijk}+\beta_{2}x_{HH\left(jk\right)}+\beta_{3}x_{Reg\left(k\right)}$ of the above equation was the fixed part of the model and $\varepsilon_{jk}+\varepsilon_{ojk}x_{ijk}+\varepsilon_{k}+\varepsilon_{ok}x_{ijk}$ is the random part of the model.

#### 2.3.4. Intra-Class Correlation Coefficient (ICC)

Individual units in a cluster often behave more alike than units from different clusters. There exists a correlation between observations when they belong to the same cluster. The amount of region and household variation is expressed as the intra-class correlation coefficient (ICC). For the three levels of binary data, the ICC is often defined for each level separately.

$$ICC_{Reg}=\frac{\sigma_{Reg}^{2}}{\sigma_{Reg}^{2}+\sigma_{HH}^{2}+\frac{\pi^{2}}{3}}\ldots \ldots ..\;ICC\;attributable\;to\;level\;3$$

$$ICC_{HH}=\frac{\sigma_{HH}^{2}}{\sigma_{Reg}^{2}+\sigma_{HH}^{2}+\frac{\pi^{2}}{3}}\ldots \ldots ..\;ICC\;attributable\;to\;level\;2$$

where $\frac{\pi^{2}}{3}$ indicates the variation of the lower (individual) level unit, $\sigma_{Reg}^{2}$ and $\sigma_{HH}^{2}$ denote the variation between region and household, respectively.

### 2.4. Ethical Approval

Ethical clearance was obtained from the Institutional Review Board of ICF International. Permission was also pursued from each woman during the fieldwork. The authors of this manuscript pursued approval from the DHS Program for use of the dataset for this study. Supplementary information about the DHS data usage and ethical standards are available at http://goo.gl/ny8T6X (accessed on 20 May 2021).

## 3. Results

### 3.1. Descriptive and Bivariate Results

The total number of children aged 6 to 59 months covered in the present study was 9461. Among these, 2602 children (about 27.5%) were undernourished at the time of the survey (Table 1). The chi-square test of association between the nutritional status of children under five and the explanatory variables in Table 1 shows that nutritional status was statistically significantly associated with the sex of the child, the age of the child, sex of the household head, birth order of the child, the mother’s education, employment status of the mother, wealth index, household size, place of residence, geographical region, the mother’s body mass index, water supply, toilet facility, childhood diarrhea and fever.

### 3.2. Multilevel Regression Results

A three-level structure (with children as a first level unit, household as a second level unit and region as a third level unit) was used. The chi-square test was used to assess heterogeneity between the regions of Ethiopia. The test results are χ^2^ = 526 with d.f = 10 (*p* = 0.001). This shows there is confirmation of heterogeneity with respect to the regions.

#### 3.2.1. Intercept-Only Model

Table 2 indicates the results of the intercept-only model. As can be seen in Table 2, ICC is used to measure the proportion of variance of child undernutrition between region and household. The ICC for the intercept only model is 0.175 and 0.257 for region and household respectively. This shows about 17.5% and 25.7% of the variation in child undernutrition is due to variations across regions and households, respectively.

#### 3.2.2. Random Intercept Model Results

Table 3 presents the three-level random intercept results and fixed-slope model. Children in the age group 16–30 months were about 21.2% (OR = 1.212) more likely to be undernourished compared to children in the age group 46–59 months. Similarly, children from a mother who had no education, primary education and secondary education were 53.9%, 23.8% and 15% more likely to be undernourished compared to women with higher education, respectively. The results of Table 3 also indicate a child between birth orders 1–3 were 13.6% (OR = 0.864) less likely to be undernourished than a child above 7 in the birth order. Employment status of the mother also had a significant effect on child undernutrition. The odds of children from an employed mother were about 0.866 times less likely to be undernourished when equated with children from an employed mother.

The incidence of undernutrition among children under five from poor families increased by 51.5%, as likened with those from rich families. The result also shows that children who live in urban areas were 31.6% less likely to be undernourished (OR = 0.684) compared to children who live in rural areas, controlling for other variables in the model. With regard to the mother’s nutritional status, children from thinner and overweight mothers were about 1.115 and 1.152 times more likely to be undernourished compared to children whose mothers had normal nutritional status, respectively.

Concerning sources of drinking water, children from households who use surface water were about 1.246 times more likely to be undernourished compared with children from households that use piped water. The results also showed that a child who had no diarrhea was 0.725 times less likely to be undernourished than a child who had diarrhea. Table 3 also shows a child who had no fever was about 16.1% less likely to be undernourished than children who had fever.

From Table 3, we see that the inclusion of level-one covariates decreased regional variations from 0.665 (level-three variance without covariates) to 0.462. This demonstrates that there is a significant variation between regions in child undernutrition. The result also shows the decrease in household variations from 0.621 (level-three variance without covariates) to 0.358, which demonstrates that there is a significant variation between regions in child undernutrition.

#### 3.3.3. The Random Coefficient Model

The results of the random slope estimates are given in Table 4. The estimated variance of wealth index was 0.052. This estimated variance indicates that there is a significant variation in the effect of wealth index across regions and household level in Ethiopia. The addition of level 1 predictors to the model results, increased the ICC (HH) = 0.235 and ICC (Reg) = 0.167, meaning that approximately 23.5% and 16.7% of the total variability in child undernutrition was due to the random factors of household and region. In Table 4 the random factors imply that there is a significant variation in the effects of wealth index; this variable fluctuates significantly across households and regions. The variance of wealth index was 0.052, which is small compared to its standard error. This supports that the effect of the wealth index may be reasonable in constraining the effect to be fixed. The correlation between the intercept and random slope of the wealth index is −0.056 and −0.042 with respect to household and region levels respectively. This implies that the incidence of undernutrition for children between aged 6–59 months from rich families was less than among those who were from poor families by a higher factor at household and region levels, with larger intercepts equated to household and regions with smaller intercepts.

#### 3.3.4. Model Comparison

The selection of an important model is a vital phase, depending on the requirement of stinginess in the model [24,25]. As seen in Table 5, the deviance value (χ^2^ = 916.54, *p* value = 0.005) is important for the intercept-only model. The deviance value (χ^2^ = 984.67, *p* value < 0.001) is important for the random intercept model which suggests that the random intercept model fits well when equated to the intercept-only model. From Table 5 AIC = 3698.12 and BIC = 3710.83, of the random intercept model, were the smallest compared to the other models considered. This suggests that the random intercept model fitted well compared to the rest of the models.

## 4. Discussion

This paper investigated the socio-economic, demographic, health and environmental factors associated with undernutrition among children aged 6–59 months in Ethiopia. This study found that place of residence, employment status of the mother, educational status of the mother, the mother’s nutritional status, age of the child, birth order of the child, source of water and having diarrhea and fever were the most important factors significantly associated with child undernutrition in Ethiopia. This finding is similar with studies conducted in Ethiopia [1,23,26,27].

The study shows, there are household- and regional-level discrepancy in undernutrition among children under five, and it is perceived that children living in rural areas of the country are at more risk of undernutrition. This result was supported by studies conducted in Ethiopia [27,28]. Consequently, it is good to support health care and food security programs in rural areas to directly address food insecurity and undernutrition problems of the low income and exposed people in the rural areas of the country. The result also showed that children of mothers with no formal education were highly exposed to undernutrition in Ethiopia. This outcome is reliable with research conducted in Ethiopia [1,26]. Therefore, it is advantageous to increase mothers’ admittance to learn in all regions in order to address the difficulty of refining their income receiving capacity and also improving the excellence of care and responsiveness they can afford to their children.

The study revealed that children from working mothers are at a greater danger of undernutrition. This finding is similar to findings of previous studies [26,28,29]. The reason for this may be that the time allotted to earn income might be at the expense of time spent in serving and caring for children, and most mothers work in the informal sectors and in lower status jobs. Therefore, it is useful to develop a policy for mothers to have sufficient time after giving birth and to provide formal and qualified jobs. Children greater than 6 months old had greater risk of undernutrition compared to other age groups. This finding supports the study conducted in Ethiopia and south Asia [9,14,15,16]. The reason for this might be that breastfeeding occurs in the initial periods of child growth. Thus, efforts should be made to communicate through different programs, such as health and nutritional training, the significance of suckling breast milk solely up to 6 months and later familiarizing other additional nutrient affluent diets.

### Strengths and Limitations

The rigorous analytical and statistical methodologies used in this study are its strength. This adds to the credibility of our work. We also present a clear methodological technique, making our research repeatable. The findings of this study can be applied to all Ethiopian children under five, because it used a nationally representative dataset. Nonetheless, our study contains some significant flaws; therefore interpretation of the results should be performed with caution. We cannot demonstrate causality between the various variables because the DHS dataset used a cross-sectional design. We can only claim associations among the studied variables.

## 5. Conclusions

The findings indicate that education, birth order, employment, wealth status, place of residence, nutritional status, source of drinking water and diarrhea are associated with undernutrition of children under five. It is therefore useful to support health care and food security programs in rural areas to directly address the food insecurity and undernutrition problems of the low-income and exposed people in rural areas of the country. The education sector must increase mothers’ admission to learning in all regions in order to overcome difficulties by refining the value of care and consideration they can give to their children. The health sector should make efforts to communicate through different programs, such as health and nutritional training, the significance of suckling breast milk solely up to 6 months and later familiarizing other additional nutrient affluent diets.

## Figures and Tables

**Table 1:** Description and bivariate results on nutritional status with explanatory variables.

Variables	Categories	Nutritional Status				Chi-Square
		Undernourished		Nourished/Normal		
		Count	%	Count	%	
Region	Tigray	136	29.1%	333	70.9%	192.62 **
	Affar	151	32%	322	68%	
	Amhara	554	39%	865	61%	
	Oromia	471	25%	1410	75%	
	Somali	213	22.4%	733	77.6%	
	Benishangul	315	37%	537	63%	
	SNNP	439	27.3%	1169	72.7%	
	Gambela	58	20%	231	80%	
	Harari	110	23%	368	77%	
	Dire Dawa	116	30.2%	268	69.8%	
	Addis Ababa	113	17%	529	83%	
Sex of child	Male	1476	30%	3444	70%	51.38 *
	Female	1135	25%	3406	75%	
Age of child in months	0–15	348	16.7%	1733	83.3%	135.46 **
	16–30	745	32.8%	1525	77.2%	
	31–45	844	34.3%	1617	65.7%	
	46–59	694	26.2%	1955	73.8%	
Sex of household head	Male	1759	28.6%	4391	71.4%	34.82 *
	Female	874	26.4%	2437	73.6%	
Birth order of the child	1–3	914	29.9%	2142	70.1%	45.75 **
	4–6	860	27.2%	2300	72.8%	
	7+	824	25.4%	2421	74.6%	
Mother’s education level	No Education	2085	38%	3402	62%	74.69 **
	Primary	1002	33.1%	2025	66.9%	
	Secondary	123	21.6%	445	78.4%	
	Higher	66	17.3%	313	82.7%	
Employment status of mother	Unemployed	1483	22.4%	5139	77.6%	98.17 **
	Employed	926	32.6%	1913	67.4%	
Wealth index	Poor	1669	42%	2304	58%	112.28 **
	Medium	809	22.5%	2786	77.5%	
	Rich	341	18%	1552	82%	
Household size	1–3	721	25.4%	2117	74.6%	32.93 *
	4–6	1574	35.4%	2873	64.6%	
	7+	472	21.7%	1704	78.3%	
Place of residence	Urban	405	21.4%	1488	78.6%	76.24 **
	Rural	2444	32.3%	5120	67.7%	
Mothers nutritional status (BMI)	Thinness	851	35.6%	1981	64.4%	86.13 **
	Overweight	306	21.5%	1398	78.5%	
	Normal	1249	25.4%	3670	74.6%	
Source of water supply	Surface water	2055	36.2%	3621	63.8%	91.54 **
	Well water	387	21.5%	1411	78.5%	
	Piped water	493	24.8%	1494	73.2%	
Type of toilet facility	Have Facilities	1350	20.4%	5272	79.6%	29.45 *
	No facilities	982	34.6%	1857	65.4%	
Had diarrhea in the two weeks before survey	Yes	1145	32.7%	2356	67.3%	74.93 **
	No	1329	22.3%	4631	77.7%	
Had fever in the two weeks before survey	Yes	1034	31.2%	2278	68.8%	45.48 *
	No	1464	23.8%	4685	72.2%	

** significant at (*p* < 0.01) and * significant at (*p* < 0.05).

**Table 2:** Results of intercept-only model.

Fixed Effect	Estimate	Std. Error	Z-Value	*p*-Value
Constant ($\beta_{0}$)	−1.863	0.530	−3.515	0.012
Random Effect				
Var (Reg)	1.665	0.334	4.985	0.008
Var (HH)	1.621	0.443	3.659	0.015
ICC (Reg)	0.175			
ICC (HH)	0.257			

**Table 3:** Results of random intercept model.

Fixed Part	Category	Coefficient	Std. Err	Z-Value	*p*-Value	Odds Ratio
Constant		−0.828	0.180	−4.6	0.0002	
Sex of child	Male	0.032	0.035	0.914	0.1804	1.056
	Female (ref)					
Age of child in months	6–15	−0.347	0.072	4.819	0.0001	0.753
	16–30	0.215	0.085	−2.529	0.039	1.212
	31–45	0.041	0.054	−0.759	0.190	0.971
	46–59 (ref)					
Sex of household head	Male	0.052	0.053	0.981	0.1633	1.125
	Female (ref)					
Birth order of the child	1–3	0.159	0.065	2.446	0.017	0.864
	4–6	−0.050	0.050	−1.000	0.1687	1.053
	7+ (ref)					
Mother’s education level	No education	−0.647	0.210	−3.081	0.012	1.539
	Primary	−0.389	0.159	−2.447	0.024	1.238
	Secondary	0.245	0.093	2.634	0.008	1.154
	Higher (ref)					
Employment status of mother	Unemployed	0.170	0.069	2.464	0.034	0.866
	Employed (ref)					
Wealth index	Poor	−0.235	0.125	−1.88	0.028	2.515
	Medium	−0.121	0.101	−1.198	0.098	1.25
	Rich (ref)					
Place of Residence	Urban	0.546	0.212	2.575	0.030	0.684
	Rural (ref)					
Mothers nutritional status (MBMI)	Thinness	0.44	0.125	3.52	0.005	1.115
	Overweight	−0.772	0.175	4.411	0.002	1.152
	Normal (ref)					
Source of water supply	Surface water	−0.087	0.035	−2.486	0.006	1.246
	Well water	0.064	0.072	0.889	0.1870	1.05
	Piped water (ref)					
Type of toilet facility	Have facilities	−0.063	0.063	−1.000	0.1587	0.674
	No facilities (ref)					
Had diarrhea in the two weeks before survey	No	−0.259	0.049	−5.286	0.001	0.725
	Yes (ref)					
Had fever in the two weeks before survey	No	−0.172	0.049	−0.351	0.0032	0.839
	Yes (ref)					
**Random part**						
Variance (Region)		0.462	0.183			
Variance (Household)		0.358	0.152			
ICC (Region)		0.154				
ICC (Household)		0.227				

ICC: Intra-Class Correlation Coefficient.

**Table 4:** Results of the random coefficient model.

Fixed Part	Category	Coefficient	Std. Err	Z-Value	*p*-Value	Odds Ratio
Constant		−0.916	0.11	−4.3	0.0012	
Sex of child	Male	0.032	0.035	0.914	0.1804	1.054
	Female (ref)					
Age of child in months	6–15	0.346	0.071	4.815	0.0002	0.751
	16–30	−0.218	0.082	−2.53	0.037	1.210
	31–45	−0.042	0.053	−0.76	0.180	0.970
	46–59 (ref)					
Sex of household head	Male	0.052	0.053	0.981	0.1633	1.128
	Female (ref)					
Birth order of the child	1–3	0.161	0.064	2.448	0.016	0.868
	4–6	−0.051	0.050	−1.02	0.1687	1.054
	7+ (ref)					
Mother’s education level	No education	−0.649	0.210	−3.083	0.013	1.534
	Primary	−0.389	0.158	−2.448	0.023	1.239
	Secondary	0.247	0.091	2.638	0.005	1.155
	Higher (ref)					
Employment status of mother	Unemployed	0.168	0.065	2.462	0.032	0.864
	Employed (ref)					
Place of residence	Urban	0.541	0.210	2.576	0.031	0.685
	Rural (ref)					
Mothers nutritional status (MBMI)	Thinness	0.442	0.124	3.564	0.003	1.112
	Overweight	−0.762	0.171	4.456	0.0012	1.154
	Normal (ref)					
Source of water supply	Surface water	−0.085	0.034	−2.5	0.006	1.248
	Well water	0.062	0.071	0.873	0.1870	1.054
	Piped water (ref)					
Type of toilet facility	Have facilities	−0.063	0.063	−1.000	0.1587	0.675
	No facilities					
Had diarrhea in the two weeks before survey	Yes	−0.257	0.048	−5.354	<0.001	0.721
	No (ref)					
Had fever in the two weeks before survey	Yes	−0.171	0.048	−0.35	0.003	0.837
	No (ref)					
**Random part**						
Variance (Region)		0. 575	0.181			
Variance (Household)		0. 483	0.154			
Variance (Wealth index)		0.052	0.054			
Covariance (Region, Household)		0.095	0.025			
Covariance (Region, Wealth index)		−0.042	0.0052			
Covariance (Wealth index, Household)		−0.056	0.0061			
ICC (Region)		0.167				
ICC (Household)		0.235				

ICC: Intra-Class Correlation Coefficient; ref: Reference category.

**Table 5:** Model comparison.

Fitted Model	Intercept-Only Model	Random Intercept Model	Random Slope (Coefficient) Model
Deviance chi-square	916.54	984.67	952.82
*p*-value	0.005	<0.000	0.001
AIC	3723.66	3698.12	3712.47
BIC	3738.20	3710.83	3721.64

AIC: Akaike information criterion; BIC: Bayesian information criterion.

## Data Availability

The dataset is available on the following website: https://dhsprogram.com/data/dataset/Ethiopia_Standard-DHS_2016.cfm?flag=0 (accessed on 20 May 2021).

## Abbreviations

LMICs	Low- and middle-income countries
EDHS	Ethiopia Demographic and Health Survey
SNNP	Southern Nations, Nationalities and Peoples
NCHS	National Centre for Health Statistics
WHO	World Health Organization
ICC	Inter class correlation coefficient
HH	Household
Reg	Region
AIC	Akaike information criteria
BIC	Bayesian information criteria
MBMI	Mother’s body mass index
